# Developmental stages of the Pekin duck (*Anas platyrhynchos f. domestica*) and the Mulard duck in artificial incubation

**DOI:** 10.1016/j.psj.2025.105132

**Published:** 2025-04-09

**Authors:** Magdalena Trela, Weronika Sołtysik, Kamil Kustra, Aleksandra Januszewska, Marcin W. Lis

**Affiliations:** a Department of Zoology and Animal Welfare, Faculty of Animal Science, University of Agriculture in Krakow Mickiewicza 21, 31-120 Kraków, Poland; b E.G.G Sp. Z o.o. sp. k.Moniuszki 15, 42-672 Wieszowa, Poland

**Keywords:** Pekin duck, Mulard duck, embryo development, development stage, hatching

## Abstract

Knowledge of the stages of avian embryo development is essential for the supervision of the incubation process in a hatchery.

The hatching eggs of Pekin (Cherry Valley) and Mulard ducks (1800 eggs/breed) were incubated at a gradually decreased temperature (from 37.8 to 36.2°C) and RH 65-60 %. Embryological sampling (3 eggs/breed/collection) was on: 0-4, 5-12 and 3- 28/32 days of incubation (d.i) every 6, 12 h and 24 h, respectively. The incubation of the remaining eggs (2 × 1600 eggs) continued until the ducklings hatched. All discarded during candling and unhatched eggs were breakout analysed.

The faster embryo development of Pekin duck in comparison to Mulard was already detected on 1 d.i. Two clear peaks of embryo mortality were observed. However, the 1st peak occurred for Pekin duck between 2 - 3 d.i. (HH 11-17), while for Mulard a critical peak in 4d.i. (HH 16-17). The 2nd peak was found for Pekin duck (17.7 % of all deaths) between 22 and 26 d.i., while for Mulard (57.3 % of all deaths) between 24 - 30 d.i. with the sharp mortality during the external pipping (29 d.i.).

From the beginning of incubation, the subsequent stages of embryonic development of the Mulard are observed later than in the Peking duck. Moreover, both breeds of ducks are characterized by the different patterns of the timeline of embryo mortality. For this reason, the incubation program needs to be targeted individually for each with these breeds.

## Introduction

Ducks, alongside chickens, are the most popular type of poultry. The global population of ducks (Anas spp.) exceeds 1.24 billion, with nearly 90 % being kept in Asia (Sumarmono, 2019; Haščík et al., 2024). Despite this large scale of production, the requirements of duck embryos and the technology of artificial incubation are much less understood compared to chickens (Cyriac et al., 2022). Meanwhile, a proper assessment of the incubation process is not possible without knowledge of the developmental stages of a given species (Fasenko, 2007; Meijerhof, 2024). In the case of the domestic chicken (Gallus gallus domesticus), embryonic development was thoroughly described in 1951 by V. Hamburger and H. L. Hamilton. This publication serves as a fundamental tool in avian embryological research. The authors divided the development of the chicken embryo into 46 stages, comprising 3 periods of embryonic development (Bellairs and Osmond, 2014).

It should be noted that 25 years after the introduction of the HH classification, a 14-stage classification of the morphogenetic development of the chicken embryo during the oviductal period (preceding egg laying) was established. Thanks to the work of Eyal-Giladi and Kochav (1976), earlier stages of development—from fertilization to egg laying—were described. Later, Sellier et al. (2006) compared the developmental stages of chicken, turkey, duck, goose, guinea fowl, and Japanese quail embryos from the fifth hour post-fertilization to the seventy-second hour of incubation.

However, there are doubts as to whether the above "keys" to recognizing the developmental stages of the chicken can be directly applied to other poultry species. Despite this, attempts to supplement knowledge in this field are very rarely made. Dupuy (2002) described the first 72 h of embryonic development of the Pekin duck. However, a detailed description of the embryonic development of the domestic duck and goose was published only in 2019 by Li et al.. Moreover, in practical settings, it is difficult to use very detailed scientific descriptions, especially since the developmental stages defined in such publications do not take into account the influence of environmental factors on the rate of embryonic development. The most important factors include:

• Factors related to the eggs obtained: egg quality and parameters (Ipek and Sozcu, 2017);
• Technological factors: handling of eggs before incubation and hatching technology (Hrncar et al., 2012);
• Microclimatic factors: temperature, air humidity, and CO2 concentration during egg storage and incubation (Liptói and Hidas, 2006);
• Factors related to the parent flock: genetic background, age, nutrition, and health status (Nasri et al., 2020).

Under their influence, the development of the embryo may be disrupted, leading to developmental deformities or its demise. Identifying the scale of losses due to embryo mortality during the aforementioned specific periods is crucial for monitoring the incubation process. For this reason, hatcheries develop simplified keys to identify the developmental stages of various bird species in relation to the incubation day under production conditions.

However, it should be emphasized that due to differences in the duration of incubation (28 days for the Pekin duck, 35 days for the Muscovy duck, and 32 days for interspecific hybrids), using guidelines that describe the embryogenesis of the domestic chicken or only one species of duck can lead to incorrect conclusions. Therefore, describing and comparing the development of the duck embryo (Pekin and Mulard), including its critical stages, seemed interesting from a scientific point of view and necessary for poultry practice.

## Material and methods

Hatching eggs came from the parent flock of Pekin ducks of the Cherry Valley meat line (1,800 pcs.) and the Mulard commercial crossbreed (1,800 pcs.) (Ł. Ostafin, Gardawice farm, Poland).

After delivery to the laboratory, the eggs were stored for 7 days at a temperature (T) of 12ºC and relative humidity (RH) of 75 %. Subsequently, the eggs were weighed, preheated to *T* = 25°C, and disinfected through ozonation (10 min at a concentration of 0.17 mg/m3, using the BT-NT18 ozone generator).

Eggs were incubated using two different programs appropriate for Pekin duck (days 1–24 of incubation: T 37.8–36.8; RH 60–65 %; days 25–29 of incubation T 36.8–36.2; RH 60–65 %); or Mulard duck (days 1–28 of incubation: T 37.8–37.0; RH 60–65 %; days 29–33 of incubation T 36.8–36.2; RH 60–65 %).

From the 10th day of incubation (E10), the eggs were cooled outside the incubators until the eggshell temperature dropped to 30-32°C. From E16, they were additionally sprayed with water at room temperature (20-22°C). Candling of the eggs was performed at E10 and during transfer to the hatching baskets. All discarded eggs and unhatched eggs were subjected to embryopathological analysis to determine the developmental phase at the time of death based on the descriptions made. Eggs not used in the embryomorphological studies (3200 eggs: 1600 Pekin duck eggs and 1600 Mulard duck eggs) were used to determine the embryo mortality curve. The obtained results are presented in the form of histograms and a linear function.

A total of 400 eggs (200 Pekin duck eggs and 200 Mulard duck eggs) were used for the analysis of embryonic development, from which 3 eggs (embryos)/sample were collected according to the scheme (Table 1) and the procedure described below (Table 2).

**Table 1:** Timeline for collecting Pekin and Mulard duck embryos for embryo-morphological descriptions.

	Pekin duck	Mulard duck
1 day	12 i 24 h incubation	24 h incubation
2-4 day	30; 36; 42; 48; 54; 60; 66; 72; 78; 84; 90; 96 h incubation	36; 48; 56; 60; 66; 72; 84; 90; 96 h incubation
5-12 day	5; 5^1^/_2_; 6; 6^1^/_2;_ 7; 7^1^/_2_; 8; 8^1^/_2_; 9; 9^1^/_2_; 10; 10^1^/_2;_ 11; 11^1^/_2_; 12; 12^1^/_2_; incubation day	5; 5^1^/_2_; 6; 8; 9; 9^1^/_2_; 10^1^/_2;_ 12; incubation day
13-32 day	13; 16; 19; 20; 21; 22; 25; 26; 27; 28 incubation day	14^1^/_2_; 16^1^/_2_; 18 ^1^/_2_; 24^1^/_2_; 30 incubation day

**Table 2:** Procedure for collecting Pekin and Mulard duck embryos for embryomorphological descriptions.

## Results

Based on the key to recognizing the development stages in chickens (HH, V. Hamburger and H. L. Hamilton, 1951), the following development stages were recognized in the Pekin duck (Table 3):

1. **12 h of incubation – Stage 2 HH:** The embryonic disc has an oval shape. In its center, a conical thickening is visible. The diameter of the embryonic disc is approximately 3-4 mm.
2. **24 h of incubation – Stage 3 or 4 HH**: **Stage 3:** The diameter of the embryonic disc increases to about 6 mm, with a clearly visible centrally located light area approximately 2 mm in diameter, surrounded by a dark field. The outer circumference of the disc forms a so-called surrounding edge about 3 mm thick. **Stage 4:** The light area changes shape to a pear-like form, about 3 mm in length, inside of which the embryo (a thin white line) is visible.
3. **30 h of incubation – Stage 6 HH:** The embryo is approximately 2-3 mm in length, with the head segment and the primitive streak clearly visible.
4. **36 h of incubation – Stage 7 or 8 HH: Stage 7:** The first pair of somites and neural folds appear in the head region. **Stage 8**: Four somites appear. Blood appears around the embryo. In both stages, the embryo has the shape of a thin line stretching across the area pellucida.
5. **42 h of incubation – Stage 9 HH**: 9 pairs of somites, primary optic vesicles appear in the head region of the embryo
6. **48 h of incubation – Stage 11 HH:** 13 pairs of somites, the heart is visible and slightly bent to the right. Body rotation appears. The embryo is approximately 7 mm in length.
7. **54 h of incubation – Stage 12 or 13 HH: Stage 12**: 16 somites – the head begins to turn to the left. **Stage 13:** 19 somites, the head is completely turned to the left, the tail bud appears, the embryo is approximately 8 mm in length..
8. **60 h of incubation – Stage 14 HH:** 22 somites are present, and at this stage, a body flexure appears. Two umbilical vessels are clearly visible. The brain vesicles are also well defined.
9. **66 h of incubation – Stage 16 HH:** 26-28 somites are present, wing buds appear, and the body flexure becomes more pronounced. The embryo is approximately 9 mm in length, and the outline of the eye begins to form. On the surface of the yolk, an increasingly prominent network of blood vessels is visible, about 3 cm in diameter, with the embryo visible as a small line in the center.
10. **72 h of incubation – Stage 17 HH:** 29-32 somites are present, leg buds appear, the flexure of the head segment remains unchanged, while the neck flexure continues to deepen.
11. **78 h of incubation – Stage 18 HH:** 30-36 somites are present, the leg buds are larger than the wing buds, the heart is visible and well vascularized, located beneath the flexed head. The network of blood vessels surrounding the embryo becomes increasingly prominent and significantly expands across the surface of the yolk. The umbilical artery and vein are particularly well defined.
12. **84 h of incubation – Stage 19 HH:** 37-40 somites are present, the leg buds are larger and more robust than the wing buds, the neck flexure forms an acute angle, and the tail is curved, pointing forward.
13. **90 h of incubation – Stage 20 or 21 HH: Stage 20:** 40-43 somites are present, pigment appears in the eye, which is approximately 1.5 mm in diameter. The embryo, about 10 mm in length, takes the shape of a comma suspended in a network of blood vessels approximately 4 cm in diameter. The heart is well vascularized. **Stage 21:** 43-44 somites are present, the wing and leg buds are enlarged and slightly asymmetrical, taking the shape of cones about 1 mm in size. The embryo is approximately 10 mm in length and 7 mm in width.
14. **96 h of incubation – Stage 22 HH:** The wing and leg buds are elongated and sharply pointed, approximately 2 mm in length. The pupil is visible, and the eye shows clear pigmentation, with the eye significantly increasing in size to about 2 mm in diameter.
15. **5 days of incubation – Stage 23 or 24 HH: Stage 23:** The wing and leg buds are approximately the same length as their width, and the contour of the back forms a curved line. **Stage 24:** The wing and leg buds are longer than they are wide, and in the leg bud, a paddle-shaped plate is visible (the toes are not yet separated). The limbs are approximately 2-3 mm in length.
16. **5 ½ days of incubation – Stage 25 HH:** Elbow and knee joints appear. The length of the upper limbs is 3 mm. The length of the lower limbs is 3.5 mm. The diameter of the eye is approximately 3 mm, and the diameter of the pupil is approximately 1 mm..
17. **6 days of incubation – Stage 26 HH:** The limbs are significantly elongated, there is a clear demarcation of the first three digits, and the beak cavity appears. The entire embryo is approximately 15 mm in length.
18. **6 ½ days of incubation – Stage 27 HH:** The formation of the beak begins; it is visible as a small, approximately 0.5 mm, cone. The length of the entire embryo is 20 mm, the eye occupies a significant portion of the head (approximately 4 mm), and the pupil is 1.2 mm. The length of the upper limb is approximately 4 mm. The length of the lower limb is approximately 5 mm
19. **7 days of incubation – Stage 28 HH**: Significant enlargement of the limbs, the digits are fused together. Significant enlargement of the beak (approximately 1 mm). The length of the upper limb is 4.5 mm. The length of the lower limb is 5.5 mm.
20. **7 ½ days of incubation – Stage 29 HH:** The wings bend at the elbows, the primordium of the fifth digit appears, and there is further growth of the beak (approximately 2 mm). The embryo is approximately 25 mm in length. The diameter of the eye is 5 mm, and the pupil is 1.5 mm.
21. **8 days of incubation – Stage 30 HH**: Three distinct segments of the wings and legs are clearly visible, with the wing bent at the elbow joint and the leg at the knee joint. Feather papillae appear, and the beak is more defined than in the previous stage. The total length of the embryo is 30 mm. The length of the upper limb is approximately 7 mm, and the length of the lower limb is approximately 8 mm.
22. **8 ½ days of incubation – Stage 31 HH:** Feather rudiments appear on arms, thighs and tail edge. Entire embryo length 33 mm. Wing length 8 mm. Leg length 9 mm, toes clearly visible, beginning to lengthen.
23. **9 days of incubation – Stage 32 HH:** The length of the embryo is approximately 35 mm; the eyelids begin to cover the eye (6 mm in diameter); the length of the wing is about 9 mm; the length of the leg is approximately 10-11 mm, with all toes clearly elongating, and differences in the size of individual toes becoming apparent.
24. **9 ½ days of incubation – Stage 33 HH:** The length of the beak is approximately 4 mm; the length of the embryo is around 38-40 mm; the length of the wing is about 10 mm; the length of the leg is 12 mm, with joints visible in the toes; feather rudiments on the tail are present in 3 rows.
25. **10 days of incubation – Stage 34 HH:** The eyes occupy a significant portion of the head (approximately 8 mm), with the eyelids covering them halfway; the length of the beak is around 5-6 mm; the total length of the embryo is 42 mm; the length of the wing is 12 mm, with feather rudiments visible along the wing's edge, and the toes continue to elongate; the length of the leg is approximately 15 mm.
26. **11 days of incubation – Stage 35 HH:** Further growth of the entire embryo occurs: phalanges appear in the digits, the beak and limbs elongate, and all feather primordia are clearly visible. The length of the embryo is 50 mm; the length of the beak is 8 mm; the length of the wing is 18 mm; the length of the leg is 22 mm.
27. **12 days of incubation – Stage 36 HH:** The length of the embryo is 60-65 mm; the length of the beak is 10 mm, with a visible egg tooth at the tip; the length of the wing is 22-25 mm; the length of the leg is 30 mm. Claw primordia are visible at the tips of the toes and on the first digit of the wing. Feather primordia cover the entire body, and the first feathers are visible on the pygostyle.
28. **13 days of incubation – Stage 37 HH**: The diameter of the eye is 8.5 mm, the eyelids cover ¾ of the eye; the length of the embryo is approximately 70 mm; the length of the wing is 30-33 mm; the length of the leg is 40 mm. The beginning of claw keratinization is visible, and the plantar pads of the foot are clearly visible. Feathers appear on both sides of the spine, on the wings, thighs, and the edge of the pygostyle.
29. **16 days of incubation – Stage 39 HH:** The embryo is fully covered with down, the claws are white and reach a length of about 2 mm. The length of the embryo is approximately 90 mm; the diameter of the eye is about 10-10.5 mm; the length of the beak is around 14 mm; the length of the wing is about 35 mm; the length of the leg is approximately 43 mm.
30. **21 and 22 days of incubation – Stage 43 HH:** Continued growth of the entire embryo; the length of the embryo is 118-120 mm.
31. **25 and 26 days of incubation – Stage 45 HH:** The extra-embryonic membranes, i.e., the yolk sac, are 50 % closed within the body cavity.
32. **27 and 28 days of incubation – Stage 46 HH:** Hatching

**Table 3:** Stages of embryonic development of Pekin duck.

Simultaneously, the applied method of evaluating the embryonic development of the Mulard duck allowed for the determination [Table 4]:

1. **24 h of incubation – Stage 1 HH:** The blastodisc is visible as a white area on the surface of the yolk and has a diameter of approximately 2-3 mm.
2. **36 h of incubation – Stage 3 HH:** The blastodisc has a circular shape and a light cream color. The diameter of the blastodisc is approximately 10 mm. The area pellucida is clearly visible in the center of the blastodisc.
3. **48 h of incubation – Stage 4 HH:** The area pellucida takes on a pear-shaped form with a length of approximately 3.5 mm, within which the embryo is located. The area opaca is expanding.
4. **56 h of incubation – Stage 9 HH:** The embryo is visible as a thin white line in the transparent area on the surface of the yolk. The embryo has 7 pairs of somites.
5. **60h incubation – Stage 11 HH:** The embryo has 13 somites, and a blood rash around the embryo is observed on the yolk ball. The embryo is approximately 7 mm in length.
6. **66h incubation – Stage 12 HH:** 16 somites, the embryo begins to twist to the left. The embryo is 8 mm in length.
7. **72h incubation – Stage 13 HH:** 19 somites, the head is fully twisted to the left, and a tail bud appears. The embryo is approximately 8 mm in length.
8. **84h incubation – Stage 14 HH:** 22 somites, a body flexure appears, and the umbilical vein becomes visible. The outline of the eye becomes visibl
9. **96h incubation – Stage 16 or 17 HH: Stage 16:** 26-28 somites, the head and body flexions deepen, the heart becomes clearly visible and assumes an "S" shape. **Stage 17:** 29-32 somites, a tail bud appears, and the heart is significantly more vascularized than in the previous stage. The embryo is 9 mm in length.
10. **5 days of incubation – Stage 19 or 20 HH: Stage 19:** 37-40 somites, the neck bend takes the shape of an acute angle, the tail is curved and directed forward. The umbilical vein and artery are clearly visible, and the network of blood vessels surrounding the embryo expands increasingly around the yolk sac. **Stage 20**: 40-43 somites, eye pigment should appear in males; females do not have eye pigment. In later stages, the eye will be visible as an outline without a filled center.
11. **6 days of incubation – Stage 22 HH:** The embryo's curvature deepens. The wing and leg buds grow and are decidedly more visible than in the previous stage. The embryo, against the background of blood vessels, is visible as an approximately 10-12 mm comma, and the network of blood vessels reaches halfway around the yolk sac.
12. **8 days of incubation – Stage 26 or 27 HH**: **Stage 26:** Limb buds elongate significantly. **Stage 27:** The process of beak formation begins, and a beak cavity appears. The embryo is approximately 15 mm in length. The eye has a diameter of about 3-4 mm.
13. **9 days of incubation – Stage 29 HH:** The embryo is suspended in a network of blood vessels that cover 3/4 of the yolk's surface. The embryo's length is 25-27 mm. The eye has a diameter of about 5 mm, the pupil is about 1.5-2 mm. The beak is about 1.5 mm in length. The wing is about 6 mm long. The leg is about 7 mm long. The knee and elbow joints are visible.
14. **10 days of incubation – Stage 30 HH:** Three distinct segments of the wings and legs are visible, the wing is bent at the elbow joint, and the leg is bent at the knee joint. The total length of the embryo is 30 mm. The upper limb length is about 7 mm. The lower limb length is about 8 mm.
15. **11 days of incubation – Stage 31 HH:** Feather primordia appear on the shoulders, thighs, and the edge of the tail. The total length of the embryo is 33 mm. The length of the wing is 8 mm. The length of the leg is 9 mm, and the digits are well visible and begin to elongate.
16. **12 days of incubation – Stage 33 HH:** The length of the beak is approximately 4-5 mm. The length of the embryo is 40 mm. The length of the wing is 10 mm. The length of the leg is 12 mm, and joints in the digits are visible. Feather primordia on the tail are arranged in 3 rows.
17. **14 days of incubation – HH stage 36:** Embryo length 60-65 mm. Beak length 10 mm, with a visible egg tooth at the tip of the beak. Wing length 25 mm. Leg length 30 mm. Rudiments of claws visible at the tips of the toes and on the first finger of the wing. Rudiments of feathers cover the entire body, with feathers visible on the pygostyle.
18. **16 days of incubation – Stage 38 HH**: The diameter of the eye is 8.5 mm. The length of the embryo is approximately 75 mm. The length of the wing is 33 mm. The length of the leg is 40 mm. The beginning of claw keratinization is visible, and the plantar pads of the foot are clearly visible. Feather growth is observed on the entire body of the embryo except for the head
19. **18 days of incubation – Stage 39 HH:** The embryo is completely covered with down. The length of the embryo is approximately 90-95 mm. The diameter of the eye is approximately 10-10.5 mm. The length of the beak is approximately 15 mm. The length of the wing is approximately 35 mm. The length of the leg is approximately 43 mm, and the claws are white and reach a length of approximately 3-4 mm.
20. **24 days of incubation – HH stage 42:** Further growth of the entire embryo body, the embryo assumes the appropriate position in the egg with the head under the right wing. Embryo length approximately 110-115 mm. Beak length approximately 17 mm. Wing length 40 mm. Leg length approximately 60 mm, with the foot measuring approximately 40 mm.
21. **30 days of incubation – HH stage 45:** Further growth of the entire embryo. Embryo length 130 mm. Wing length approximately 50 mm. Leg length approximately 68 mm, with the foot measuring approximately 40 mm.

**Table 4:** Stages of embryonic development of Mulard duck.

### *Distribution of mortality of Pekin and Mulard duck embryos during incubation*

**Figure 1:**
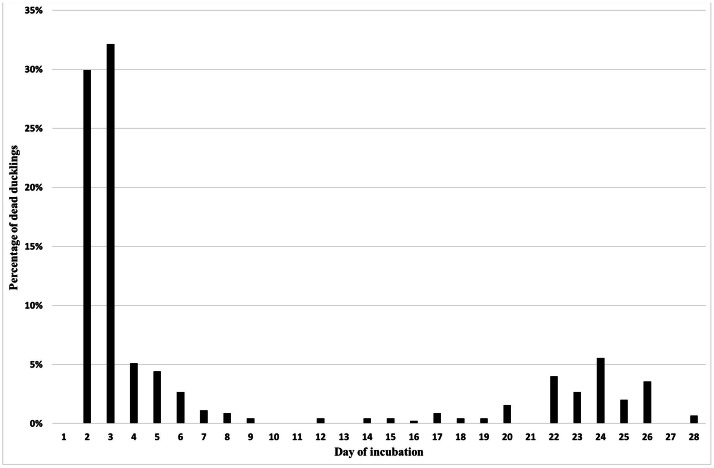
Distribution of mortality of Pekin duck embryos during incubation.

In the case of the Pekin duck, two distinct peaks of mortality were observed. The first one, during which 74.3 % of all embryos died, lasted between the 2nd and 6th day of incubation with a critical period on the 2nd-3rd day of incubation. This corresponded to stages 11 to 17. The second peak of mortality began on the 22nd and ended on the 26th day of incubation. During this period, 17.7 % of all embryos died. It is difficult to clearly determine the second critical period, but it seems to fall on the 24th-26th day of incubation, i.e. the internal and external piercing phases (Figure 1).

**Figure 2:**
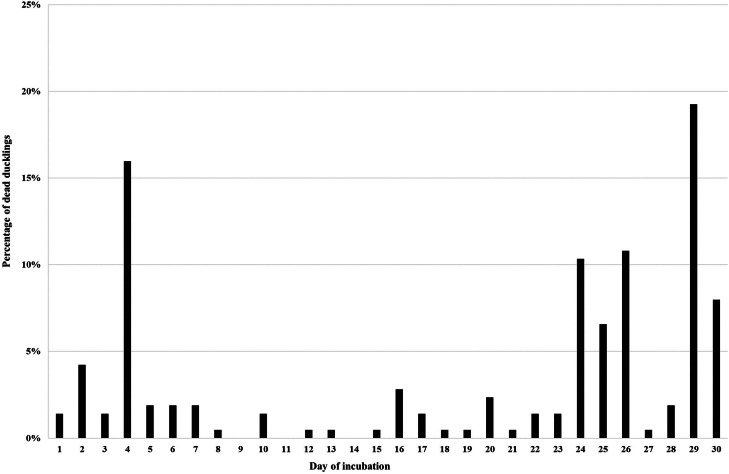
Distribution of mortality of Mulard duck embryos during incubation.

In the case of the Mulard duck, two distinct peaks of mortality were observed. The first one, during which 28.6 % of all embryos died, occurred between days 2 and 7 of incubation, with the critical period falling on day 4 of incubation. This corresponded to stages 16 and 17. The second peak of mortality began on day 24 and ended on day 30 of incubation. During this period, 57.3 % of all embryos died, and the critical period falls on day 29 of incubation, which is the phase of external pipping (Figure 2).

## Discussion

Currently, the production of chicks of various poultry species is carried out on a massive scale. Errors made at different stages of the technological process significantly impact hatching results. To accurately diagnose the cause of embryo mortality, a thorough embryopathological analysis must be performed, which is not possible without knowledge of the successive stages of embryonic development of a given bird species. For this reason, many descriptions of developmental stages have been created for different bird species (Nagai et al., 2011; Hemmings et al., 2016; Łukaszewicz et al., 2019; Mueller et al., 2022; Cordero and Werneburg, 2022), but they are lacking for Muscovy ducks, and especially for interspecific hybrids.

The primary tool in poultry embryopathological studies is the detailed description of the developmental stages of the domestic chicken, compiled by V. Hamburger and H. L. Hamilton in 1951. The developmental phases described under laboratory conditions do not exactly align with the incubation days assigned to them in practice. Furthermore, the incubation period of eggs from different poultry species varies, leading to obvious discrepancies in the interpretation of results. A study conducted by Lumsangkul et al. (2018) revealed differences in developmental stages between Tsaiya ducks and Taiwanese chickens. They observed that duck embryos develop approximately 16 h slower than chicken embryos during the first 72 h of development. After 72 h of incubation, 40 somites were observed in the Tsaiya duck embryo, while only 32 somites were present in the Taiwanese chicken embryos. The onset of limb bud development in the duck occurred at 51 h after the start of incubation, whereas in chicken embryos, it occurred only at 64 h of incubation. In contrast, Dupuy et al. (2002) demonstrated that the early morphogenetic development of the Pekin duck is comparable to that of the chicken. However, the duck embryo is less developed than the chicken embryo at the time of egg laying. They also noted that Koller's sickle is visible only in chicken embryos.

The conducted studies confirm the above opinions, with differences being noticeable even in the early stages of embryonic development. In the case of the birds included in the study, the standard incubation period for Pekin ducks is 28 days, while for the interspecies hybrid – Mulard – it is 30-32 days. This results in a slower development of Mulard duck embryos. For example, Pekin duck embryos were classified at stage 12 HH between the 51st and 55th hours of incubation (for 4 h), whereas Mulard ducks were classified between the 66th and 72nd hours of incubation (for 6 h) (Table 5). Li et al. (2019) also observed differences in the rate of embryonic development already in the period after egg laying. After 72 h of egg storage, Mulard embryos were classified as stage VII, while Pekin duck embryos were classified as stage VIII (Eyal-Giladi and Kochav, 1976). It should also be noted that determining a precise key to define the individual developmental stages is extremely difficult. Often, when collecting embryos in one sample, several developmental phases were prepared, but never more than 3 stages. For example, stages 19 and 20 for the Mulard hybrid occurred on the same incubation day (the 5th day). A similar situation is observed in the embryonic development of the domestic chicken, where, for instance, stage 19 can be identified between the 68th and 72nd hours, and the subsequent stage 20, between the 70th and 72nd hours of incubation (Hamburger and Hamilton, 1951). At the same time, the results obtained in the discussed own studies don't differ from those described by Li et al. (2019).

**Table 5:** Comparison of embryonic development stages according to the Hamburger and Hamilton (1951; Bellairs and Osmond, 2014) key for the chicken, Pekin and Mulard duck during the subsequent stages of incubation (h – hour of incubation; D – day of incubation).

Stage HH	Chicken	Pekin duck	Mulard duck
	hour/day incubation		
1	0-6 h	0-11 h	24 h
3	12-13 h	18-20 h	36 h
4	18-19 h	24-26 h	48 h
9	29-33 h	39-42 h	56 h
11	40-45 h	46-52 h	60 – 66 h
12	45-49 h	51-55 h	66 - 72
13	48-52 h	54-58 h	72 h
14	50-53 h	56-59 h	84 h
16	51-56 h	62-69 h	96 h
17	52-64 h	67-78 h	96 h
19	68-72 h	81-87 h	5 D
20	70-72 h	85-91 h	5 D
21	3,5 D	90-95 h	5.5 D
22	3,5 D	4 D	6 D
26	4,5-5 D	6 D	8 D
27	5 D	6,5 D	8 D
29	6 D	7,5 D	9 D
30	6,5 D	8 D	9,5 D
31	7 D	8,5 D	10,5 D
33	7,5-8 D	9,5-10 D	12 D
36	10 D	11,5-12,5 D	14,5 D
38	12 D	14-15 D	16,5 D
39	13 D	16 D	18,5 D
42	16 D	19-20 D	24,5 D
45	19-20 D	25-26 D	30 D
46	20-21 D	27-28 D	31-32 D

Precise identification of the early developmental stages allows for a more accurate analysis of eggs during the first candling. Brun et al. (2004) found no differences in early embryo mortality (2 to 3 %) between Mulard duck embryos and Pekin duck embryos during candling after 6 days of incubation. However, a detailed embryopathological analysis revealed early embryo mortality rates of 14.9 % for the Mulard and 10.3 % for the Pekin duck. These results directly reflected the condition of the parent flock and the errors made in the initial phase of incubation.

A very important aspect of egg incubation technology is determining the periods of embryo mortality. A thorough understanding of mortality curves for different poultry species and the subsequent identification of the developmental stage at which the embryo died has a significant impact on optimizing this technological process. Avian embryos are more likely to die during the early and late stages of development (Hemmings et al., 2016). In the domestic chicken, the so-called first peak of embryo mortality occurs between days 1 and 7 of incubation, with the critical period typically occurring between days 3 and 5 of incubation, which corresponds to stages 12-25 of the HH classification (Romanoff, 1949; Romanoff and Romanoff, 1972; Borzemska and Janowski, 1984; Fasenko, 2007; Bellairs, 2014). In the presented studies for Pekin duck embryos, the first peak of mortality was determined between the 2nd and 6th day of incubation with a critical period falling on the 2nd-3rd day of incubation. In contrast, for the Mulard hybrid, the peak of mortality was observed between the 2nd and 7th day of incubation with a critical period on the 4th day of incubation. Interestingly, in turkeys, whose egg physical characteristics and incubation period are similar to those of Pekin ducks (egg mass 70-90 g and 28 days), the critical period occurs only on the 5th day of incubation (French, 2000; Mróz et al., 2010). The second peak of mortality in the artificial incubation of birds occurs during the hatching period. For the domestic chicken, this is on days 19-20 of incubation (Borzemska and Janowski, 1984), and for turkeys, it is on days 25-27 of incubation (French, 2000; Mróz et al., 2010). In the case of ducks, the critical period appears to be more extended, as increased mortality was observed between the 22nd and 26th days of incubation in Pekin ducks and between the 24th and 30th days in Mulard ducks.

In summary, the individual stages of embryonic development last longer in the Mulard duck compared to the Pekin duck. Differences are already noticeable in the initial stages of incubation. The first peak of embryo mortality during Pekin duck incubation occurs between the 2nd and 6th days of incubation, with the critical period observed on the 2nd and 3rd days. In contrast, for Mulard ducks, the peak occurs between the 2nd and 7th days of incubation, with the critical period on the 4th day of incubation. The second peak of mortality in the Pekin duck was observed between the 22nd and 26th day of incubation (incubation), and in the Mulard duck between the 24th and 30th day of incubation. The critical period in both species was related to the effort required for internal and external puncture.

## Disclosures

The authors declare that they have no known competing financial interests or personal relationships that could have appeared to influence the work reported in this paper.
